# Supplementary material for: TREM-1 links dyslipidemia to inflammation and lipid deposition in atherosclerosis
Source: Nat Commun. 2016 Oct 20;7:13151. doi: 10.1038/ncomms13151 (PMC5080444; doi:10.1038/ncomms13151)
Supplement: Supplementary Information — Supplementary Figures 1-7, Supplementary Tables 1-4 and Supplementary References. [file ncomms13151-s1.pdf]

Supplementary Figure 1

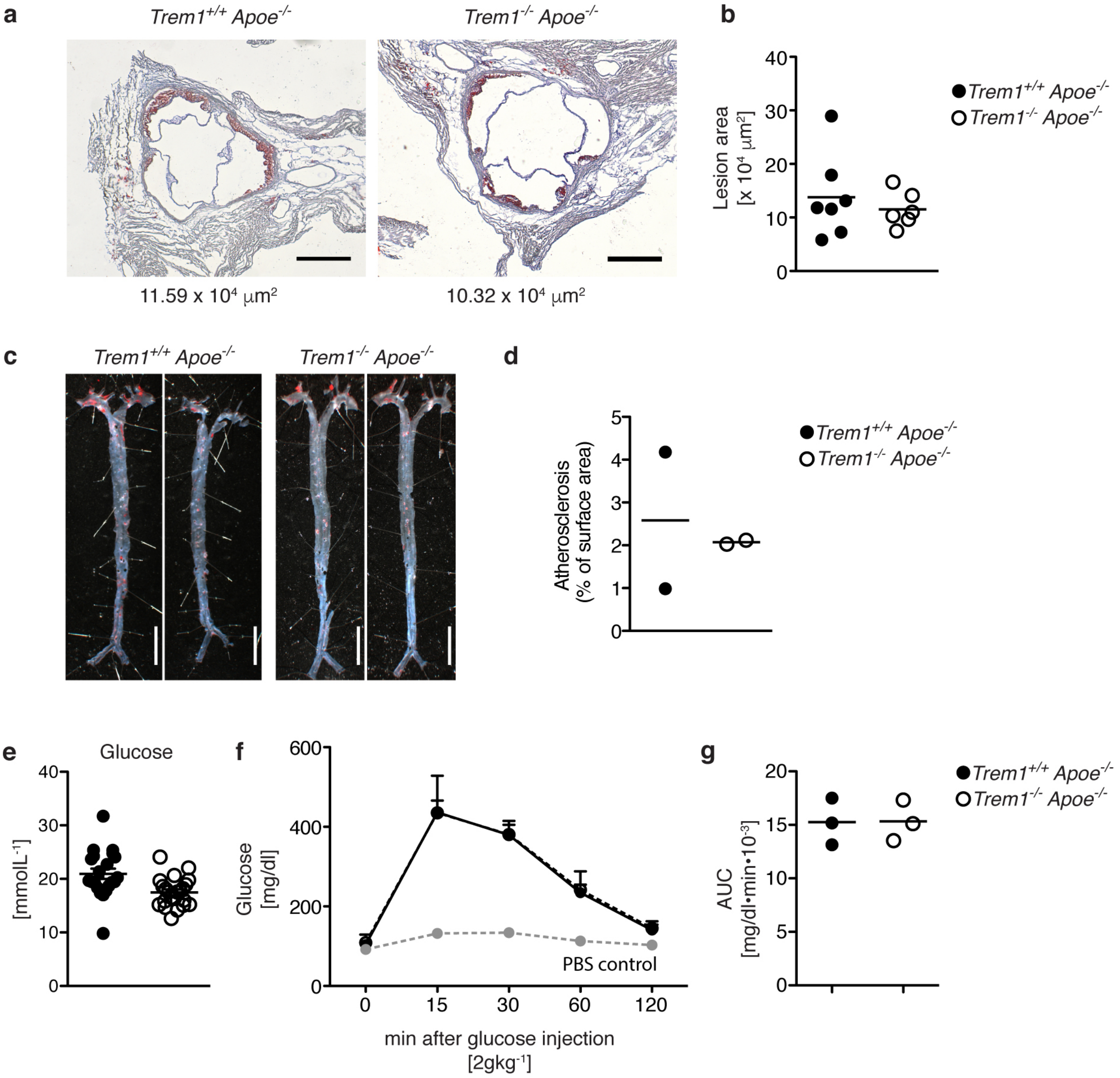

Supplementary Figure 1

(a-d) Analysis of aortic sinus sections at 4 weeks post HFCD or of aortas derived from 16 week chow-fed mice does not indicate a major impact of TREM-1 during early stages of atherogenesis. (a,b) Extent of atherosclerosis in the aortic root at 4 weeks post HFCD feeding. (**a**) Representative examples of ORO stained sections of the aortic sinus. Scale bars indicate 500 µm. (**b**) The lesion area was calculated from 10 sequential ORO stained sections for each *Trem1*$^{+/+}$ *Apoe*$^{-/-}$ and *Trem1*$^{-/-}$ *Apoe*$^{-/-}$ mouse. Circles show data for individual mice. **(c,d)** Extent of atherosclerotic lesions in aortas of 16 week chow-fed mice (n=2 per group). **(c)** *en face* preparations of ORO-stained aortas. Scale bars indicate 10 mm. **(d)** Overall extent of atherosclerosis (aortic lesion surface area expressed as % of total aortic surface).

(e-g) TREM-1 does not impact on serum glucose levels or glucose clearance in HFCD-fed mice. **(e)** Fasting serum glucose concentrations in *Trem1*$^{+/+}$ *Apoe*$^{-/-}$ (n=13) and *Trem1*$^{-/-}$ *Apoe*$^{-/-}$ (n=12) mice at 16 weeks post HFCD-feeding were determined on a cobas 8000 clinical chemistry analyzer (Roche diagnostics). (**f,g)** Intraperitoneal glucose tolerance test (IPGTT). 16 week HFCD-fed *Trem1*$^{+/+}$ *Apoe*$^{-/-}$ and *Trem1*$^{-/-}$ *Apoe*$^{-/-}$ mice were fasted o/n and challenged i.p. with 2 mg/kg glucose. (**f**) Blood glucose levels were calculated from blood samples obtained at the indicated intervals from the lateral tail vein using an Accu Chek blood glucose meter (Roche diagnostics). Mean values for n=3 mice per group are shown with error bars indicating the SD. (**g**) Area under the curve (AUC) of IPGTT as shown in (**f**). Statistical testing employed the two-tailed t-test. Statistically not significant differences with $p > 0.05$ are not indicated.

Supplementary Figure 2

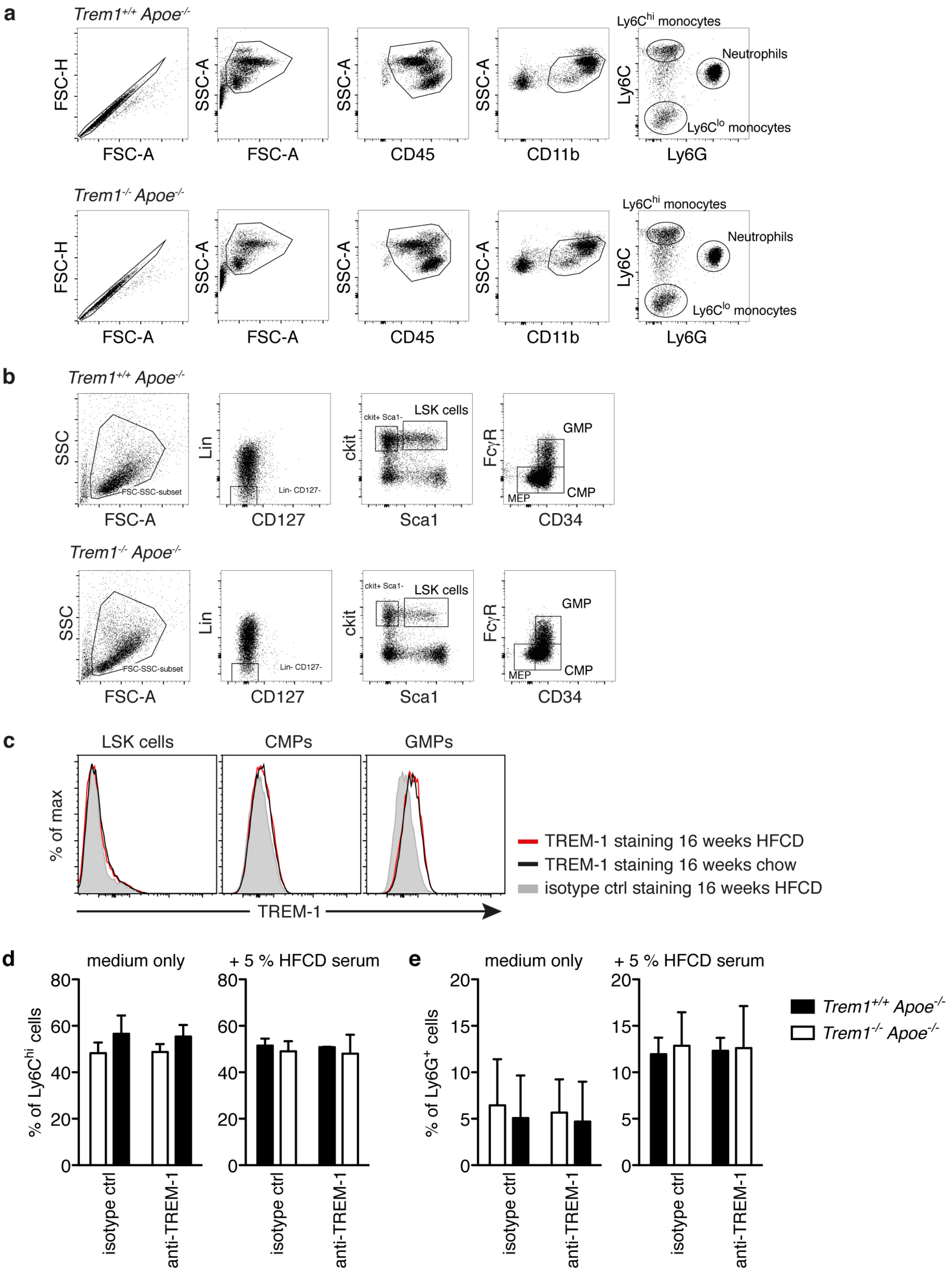

Supplementary Figure 2

(a-c) Gating strategies and representative staining panels for peripheral blood myeloid cell subsets and bone marrow hematopoietic stem and progenitor cells.

(a) Gating strategy and representative dot plots for peripheral blood myeloid cell subsets in 16 week HFCD-fed *Trem1*$^{+/+}$ *Apoe*$^{-/-}$ and *Trem1*$^{-/-}$ *Apoe*$^{-/-}$ mice. **(b)** Gating strategy and representative dot plots for identification of hematopoietic stem cell-enriched LSK cells and myeloid progenitor cells in lineage marker negative (lin^{-}) CD127^{-} bone marrow (BM) cells of 16 week HFCD-fed *Trem1*$^{+/+}$ *Apoe*$^{-/-}$ and *Trem1*$^{-/-}$ *Apoe*$^{-/-}$ mice. LSK cells were identified by their Sca1^{+}ckit^{hi} phenotype while GMP and CMP were discriminated among the Sca^{-} ckit^{hi} population based on their expression levels of FcγR and CD34. **(c)** Representative histogram overlays showing TREM-1 surface expression (bold line) in LSK cells, CMP and GMP as defined in **(b)** in 16 week HFCD-fed (red line) versus chow-fed (black line) *Trem1*$^{+/+}$ *Apoe*$^{-/-}$ mice. Filled histograms represent matched isotype control stained cells from HFCD-fed mice.

(d,e) TREM-1-mediated stimulation of GMP isolated from 16 week HFCD-fed *Trem1*$^{+/+}$ *Apoe*$^{-/-}$ mice does not augment monocyte differentiation *in vitro*. **(d,e)** GMP were FACS-sorted from 16 week HFCD-fed *Trem1*$^{+/+}$ *Apoe*$^{-/-}$ and *Trem1*$^{-/-}$ *Apoe*$^{-/-}$ mice and cultured in 96-well U-bottom plates in IMDM 10% FCS supplemented with IL-11 and TPO (10 ng/ml) as well as SCF and FLT3L (50 ng/ml) in the presence of plate-bound anti-TREM-1 or isotype control mAb (10 μg/ml). Where indicated, the medium was additionally supplemented with 5% HFCD serum. After 72 h of culture, cells were analyzed by flow cytometry for the expression of monocytic and granulocytic lineage markers. **(d)** Relative frequency of Ly6C^{hi} monocytes. **(e)** Relative frequency of Ly6G^{+} neutrophils. Columns show mean values of n=6 mice per group from two independent experiments with error bars indicating the SD. (Conditions with 5% HFCD serum: mean of n=2 mice from one experiment with error bars indicating the range).

Supplementary Figure 3

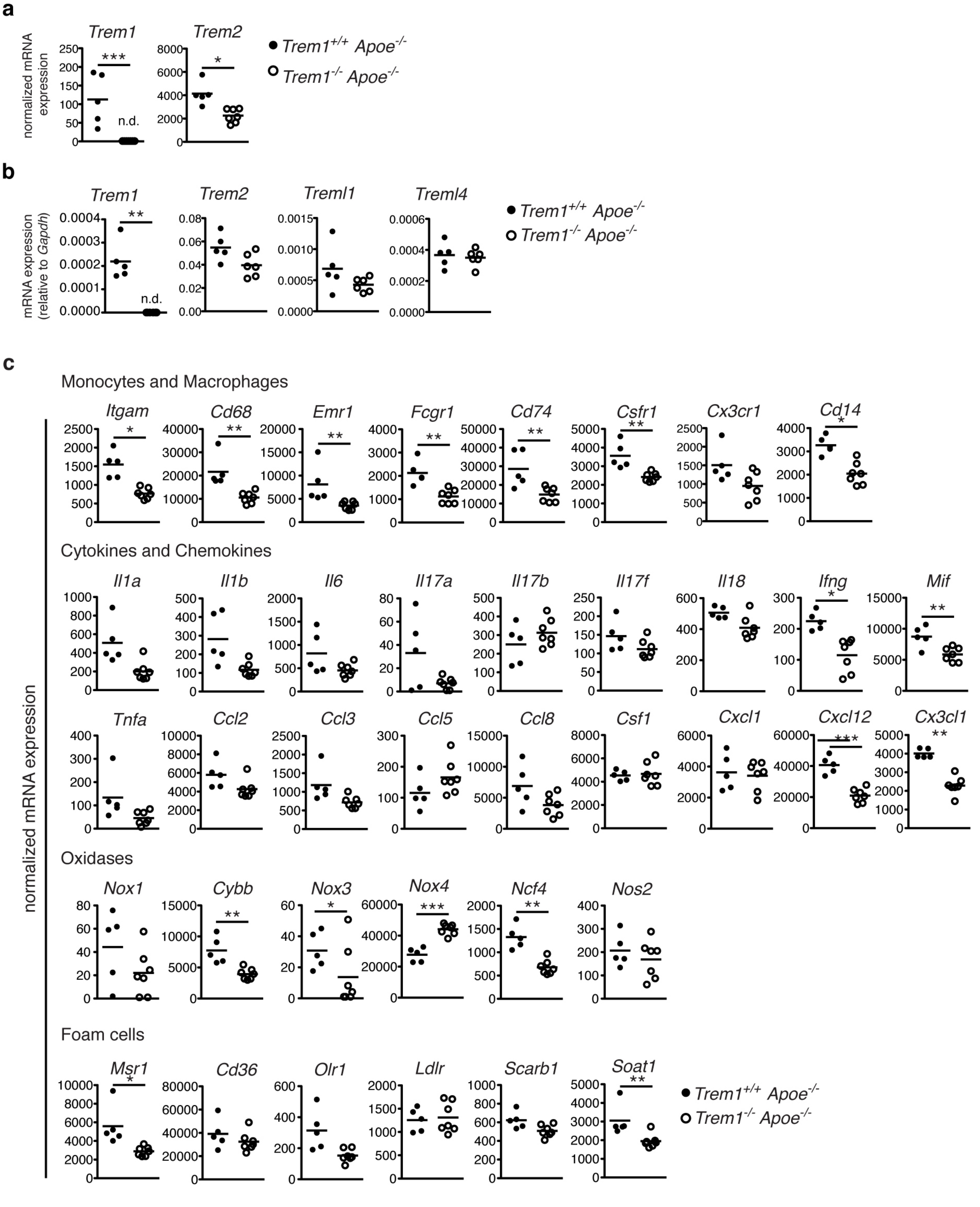

Supplementary Figure 3

Expression of TREM family members and selected genes of interest in the aortic root of 16 week HFCD-fed *Trem1*$^{+/+}$ *Apoe*$^{-/-}$ *versus* *Trem1*$^{-/-}$ *Apoe*$^{-/-}$ mice.

(a) Nanostring nCounter-based quantification of *Trem1* and *Trem2* mRNA expression, nd: not detected. **(b)** qRT-PCR-based quantification of *Trem1*, *Trem2*, *Trem1l* and *Trem4l* mRNA. **(c)** Nanostring nCounter-based mRNA counts of selected genes of interest. Symbols show individual values for 16 week HFCD-fed *Trem1*$^{+/+}$ *Apoe*$^{-/-}$ (n=5) and *Trem1*$^{-/-}$ *Apoe*$^{-/-}$ (n=7) mice. * $p < 0.05$, ** $p < 0.01$, *** $p < 0.001$. Significant differences in gene expression were calculated as described in the Methods section. Statistically not significant differences with $p > 0.05$ are not indicated.

Supplementary Figure 4

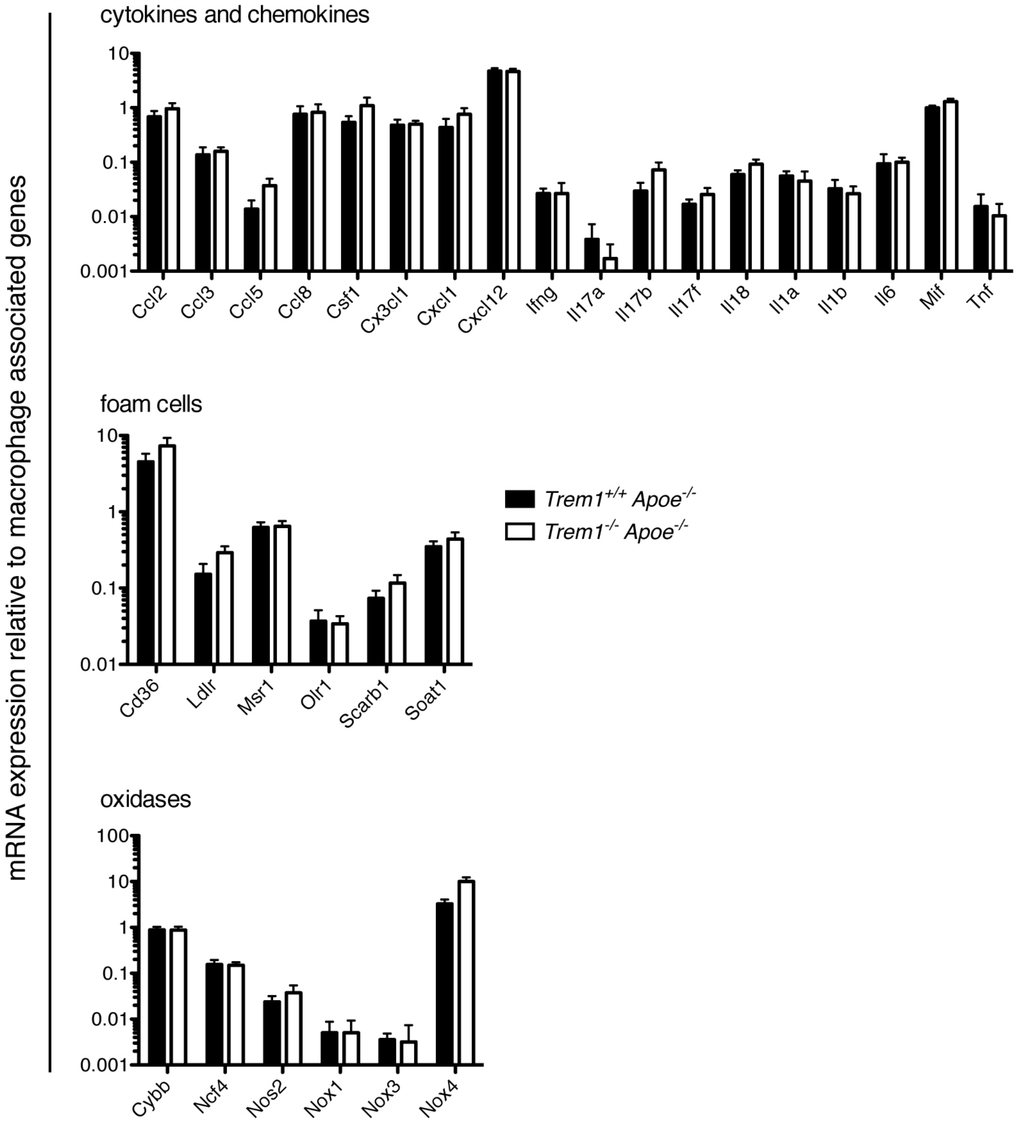

Supplementary Figure 4

Nanostring-based gene expression data after normalization to combined macrophage markers. Expression levels of *Cd14, Cd68, Cd74, Csf1r, Cx3cr1, Emr1, Fcgr1* and *Itgam* were used to calculate a mean combined macrophage marker expression level. For each sample, the expression of a candidate gene was calculated relative to the mean macrophage marker expression level. Column graphs show mean mRNA expression levels of genes of interest relative to macrophage-associated gene expression for each group of $Trem1^{+/+}$ $Apoe^{-/-}$ (n=5) and $Trem1^{-/-}$ $Apoe^{-/-}$ (n=7) mice. Bars indicate mean + SD.

Supplementary Figure 5

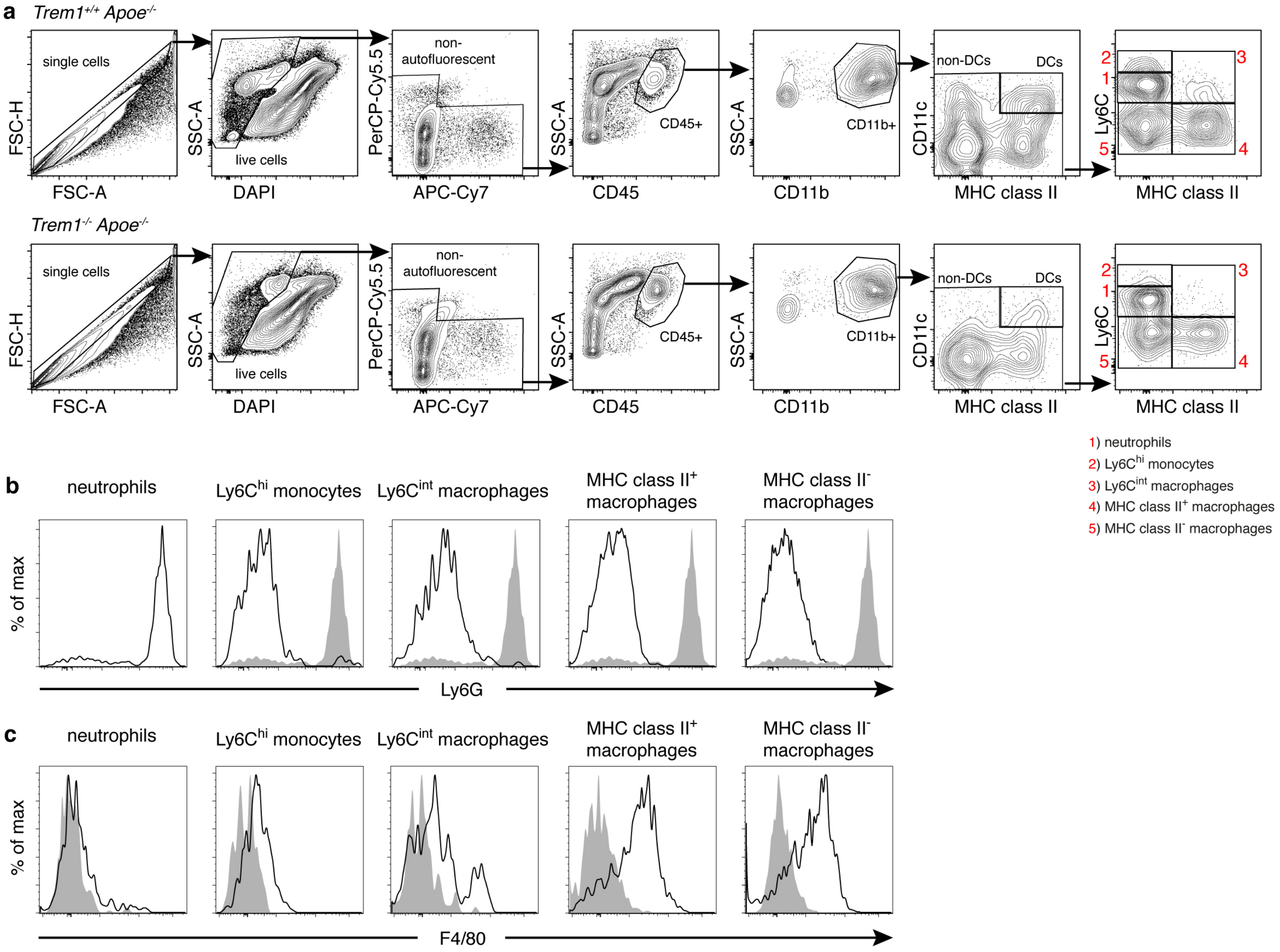

Supplementary Figure 5

Gating strategy for identification of aortic wall-infiltrating myeloid cell subsets.

(a) Gating strategy for identification of aortic wall-infiltrating myeloid cell subsets and representative contour plots for cells retrieved from the digested aortas of 16 week HFCD-fed *Trem1*$^{+/+}$ *Apoe*$^{-/-}$ and *Trem1*$^{-/-}$ *Apoe*$^{-/-}$ mice. Initial gating comprised the exclusion of doublets, dead (DAPI^{+}) and autofluorescent cells. Autofluorescence was defined based on double-positive expression of Ly6G (PerCP-Cy5.5) and MHC class II (APC-Cy7). Among single, live and non-autofluroescent cells leukocytes were subsequently discriminated from endothelial and stromal cells by gating on CD45^{+} cells. CD45^{+} leukocytes were further subgated into CD11b^{+} cells. Among CD11b^{+} cells, we gated out MHC class II^{+} CD11c^{+} cells as these were reported to represent dendritic cells (DC) [1,2]. The remaining CD11b^{+} cells were finally separated according to their expression levels of Ly6C and MHC class II to

distinguish five myeloid cell subsets: 1. Neutrophils ($Ly6C^{int}$, $MHCII^{-}$), 2. $Ly6C^{hi}$ monocytes ($Ly6C^{hi}$, $MHCII^{-}$) 3. $Ly6C^{int}$ macrophages ($Ly6C^{int}$ $MHCII^{+}$) 4. $MHCII^{+}$ macrophages ($Ly6C^{lo}$ $MHCII^{+}$) and 5. $MHCII^{-}$ macrophages ($Ly6C^{lo}$, $MHCII^{-}$). (**b)** Ly6G surface expression of aortic wall infiltrating myeloid cell subsets. Black lines show Ly6G expression of the indicated subsets while gray filled histograms indicate Ly6G expression of neutrophils in comparison. **(c)** F4/80 expression of aortic wall-infiltrating myeloid cell subsets. Black lines show F4/80 expression of the indicated subsets with gray filled histograms representing isotype controls.

Supplementary Figure 6

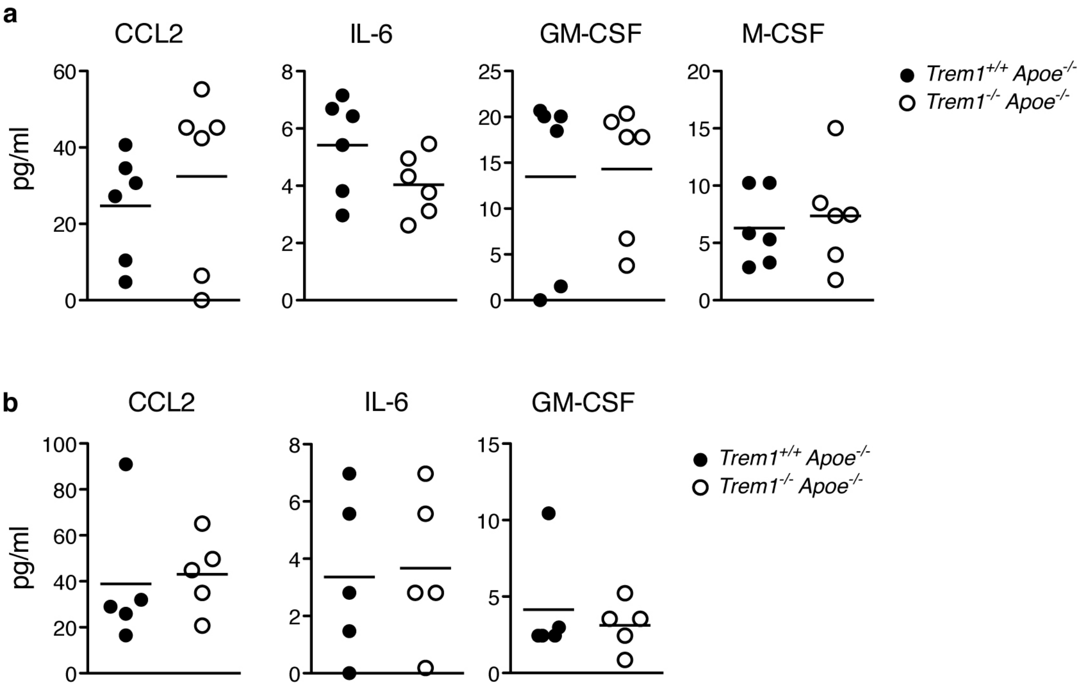

Supplementary Figure 6

TREM-1 has no appreciable impact on the level of distinct cytokines and chemokines.

(a) Bone flushes and **(b)** sera derived from *Trem1*$^{+/+}$ *Apoe*$^{-/-}$ and *Trem1*$^{-/-}$ *Apoe*$^{-/-}$ mice at 16 weeks post HFCD feeding were analyzed using either **(a)** the mouse discovery cytokine and chemokine 31-plex assay (Eve Technologies, Canada) or **(b)** a Luminex-based mouse cytokine 20-plex assay (LMC0006, Thermo Fisher Scientific) (M-CSF not included in assay). Symbols show individual values for 16 weeks HFCD-fed *Trem1*$^{+/+}$ *Apoe*$^{-/-}$ (n=5) and *Trem1*$^{-/-}$ *Apoe*$^{-/-}$ (n=5) mice with lines indicating mean values per group. Statistically not significant differences with $p > 0.05$ are not indicated.

Supplementary Figure 7

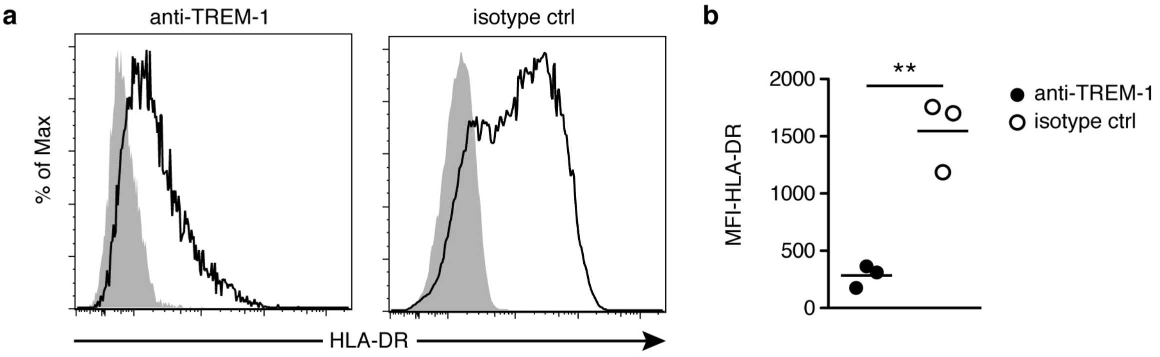

Supplementary Figure 7

TREM-1-mediated signaling reduces HLA-DR expression during macrophage differentiation *in vitro*.

Primary human monocytes were differentiated to macrophages with recombinant human M-CSF (50 ng/ml) in the presence of a plate-bound agonistic anti-TREM-1 antibody or an isotype control antibody. After 4 days of culture, macrophages were detached, stained for HLA-DR and analyzed by flow cytometry by gating on single live cells. **(a)** Representative histograms of HLA-DR expression. Black lines represent the HLA-DR-stained cells and gray area show matched isotype control-stained cells. **(b)** MFI values for HLA-DR surface expression (with subtracted MFI values of matched isotype control-stained cells). Circles represent data for individual blood donors, lines indicate mean values of three independent experiments . ** $p<0.01$ as determined by the two-tailed t test.

Supplementary Table 1
List of genes added to the NanoString mouse immunology panel

RefSeqID	symbol	logFC	adj.P.Val
NM_013454.3	***Abca1***	-0.548182	0.020914
NM_009593.1	***Abcg1***	-0.903426	0.007975
NM_007482.3	***Arg1***	-1.605327	0.483673
NM_009754.3	***Bcl2l11***	-0.656498	0.032398
NM_009853.1	***Cd68***	-1.021322	0.008107
NM_009141.2	***Cxcl5***	-0.516252	0.733208
NM_007837.3	***Ddit3***	-0.098331	0.513715
NM_010439.3	***Hmgb1***	0.084819	0.668339
NM_010637.3	***Klf4***	-0.103353	0.738274
NM_010700.2	***Ldlr***	0.047427	0.913072
XM_909927.2	***Ly6g***	-1.953349	0.250607
NM_008562.3	***Mcl1***	-0.083424	0.716881
NM_008587.1	***Mertk***	-0.022212	0.922680
NM_008605.3	***Mmp12***	-0.599649	0.238986
NM_010902.3	***Nfe2l2***	-0.108595	0.573927
NM_145827.3	***Nlrp3***	-0.803574	0.284296
NM_009473.2	***Nr1h2***	-0.003419	0.993634
NM_013839.2	***Nr1h3***	-0.361939	0.088568
NM_011851.3	***Nt5e***	-0.057942	0.909367
NM_008744.2	***Ntn1***	-0.133649	0.519637
NM_138648.1	***Olr1***	-0.996517	0.470639
NM_008920.4	***Prg2***	-0.046406	0.887030
NM_016741.1	***Scarb1***	-0.287325	0.093439
NM_011355.1	***Sfpi1***	-0.809824	0.010667
NM_145581.1	***Siglec5***	-1.569313	0.189712
NM_009230.3	***Soat1***	-0.629950	0.006853
NM_146064.1	***Soat2***	-0.994748	0.496803
NM_011480.1	***Srebf1***	-0.402164	0.063383
NM_011604.3	***Tlr6***	-0.710518	0.012207
NM_001025250.3	***Vegfa***	-0.577743	0.007533

Supplementary Table 2

List of genes that were significantly differentially expressed (adj. p. val < 0.05) between 16 weeks HFCD-fed *Trem1* $^{+/+}$ *Apoe* $^{-/-}$ vs. *Trem1* $^{-/-}$ *Apoe* $^{-/-}$ mice

RefSeqID	symbol	logFC	adj.P.Val
NM_021406.3	***Trem1***	-6.548570	0.000589
NM_013545.2	***Ptpn6***	-1.315980	0.000903
NM_021704.3	***Cxcl12***	-0.970956	0.000903
NM_007572.2	***C1qa***	-0.912202	0.001407
NM_001110323.1	***Klra7***	-3.782532	0.001423
NM_009851.2	***Cd44***	-0.766548	0.001423
NM_008528.4	***Blnk***	-1.140291	0.002526
NM_008397.3	***Itga6***	-0.993086	0.003698
NM_008677.2	***Ncf4***	-0.979928	0.003698
NM_007807.2	***Cybb***	-0.962905	0.003698
NM_010233.1	***Fn1***	-0.749615	0.003698
NM_007486.4	***Arhgdib***	-0.730459	0.003698
NM_010130.1	***Emr1***	-1.094794	0.004017
NM_009777.2	***C1qb***	-0.943235	0.004017
NM_009230.3	***Soat1***	-0.629950	0.006853
NM_031178.2	***Tlr9***	-1.117207	0.007283
NM_010186.5	***Fcgr1***	-0.947866	0.007283
NM_001042605.1	***Cd74***	-0.926043	0.007283
NM_011577.1	***Tgfb1***	-0.601625	0.007283
NM_011610.3	***Tnfrsf1b***	-0.932845	0.007533
NM_009369.4	***Tgfbi***	-0.644661	0.007533
NM_008404.4	***Itgb2***	-1.002995	0.007630
NM_010185.4	***Fcer1g***	-1.104990	0.007717
NM_009142.3	***Cx3cl1***	-0.841099	0.007717
NM_009593.1	***Abcg1***	-0.903426	0.007975
NM_009853.1	***Cd68***	-1.021322	0.008107
NM_001113474.1	***Lair1***	-1.077760	0.008895
NM_011662.2	***Tyrobp***	-0.987466	0.008895
NM_001111021.1	***Runx1***	-0.895071	0.008895
NM_010378.2	***H2-Aa***	-0.806005	0.009241
NM_001170632.1	***Fcamr***	-3.296913	0.009272
NM_001033122.3	***Cd69***	-3.300441	0.009388
NM_008604.3	***Mme***	-0.884249	0.009915
NM_010382.2	***H2-Eb1***	-1.015780	0.010136
NM_011355.1	***Sfpi1***	-0.809824	0.010667
NM_021281.2	***Ctss***	-1.030240	0.011031
NM_013640.3	***Psmb10***	-0.688050	0.011031
NM_001082960.1	***Itgam***	-1.004610	0.011920
NM_011604.3	***Tlr6***	-0.710518	0.012207
NM_001271430.1	***Cd82***	-0.607500	0.012303
NM_010745.2	***Ly86***	-0.762518	0.015393
NM_011638.3	***Tfrc***	-1.094356	0.016295
NM_001038604.1	***Clec5a***	-1.069723	0.016295
NM_008348.2	***Il10ra***	-1.138435	0.016696
NM_012057.3	***Irf5***	-0.666845	0.018486
NM_031254.2	***Trem2***	-0.895160	0.019268
NM_007649.4	***Cd48***	-1.118095	0.019909
NM_001043317.2	***Cd22***	-1.316593	0.020476
NM_011210.3	***Ptprc***	-0.843256	0.020486
NM_207105.2	***H2-Ab1***	-0.812375	0.020486
NM_001037177.1	***Nfatc2***	-0.849600	0.022073
NM_008873.2	***Plau***	-0.791118	0.022073
NM_001077189.1	***Fcgr2b***	-0.733583	0.022895
NM_009856.2	***Cd83***	-0.622932	0.022895
NM_013482.2	***Btk***	-0.921450	0.023260
NM_008250.2	***Hlx***	-0.778719	0.023930

RefSeqID	symbol	logFC	adj.P.Val
NM_009914.4	***Ccr3***	-3.754729	0.026021
NM_010188.5	***Fcgr3***	-0.712803	0.026021
NM_009911.3	***Cxcr4***	-0.992579	0.027832
NM_001113326.1	***Msr1***	-0.901083	0.028592
NM_011827.3	***Hcst***	-1.752307	0.029092
NM_010576.3	***Itga4***	-0.628088	0.029092
NM_198958.2	***Nox3***	-2.618617	0.030037
NM_011905.2	***Tlr2***	-0.608692	0.030770
NM_019494.1	***Cxcl11***	-2.505075	0.032061
NM_011095.2	***Lilrb3***	-0.968129	0.032061
NM_009754.3	***Bcl2l11***	-0.656498	0.032398
NM_008337.1	***Ifng***	-1.147221	0.032478
NM_011693.2	***Vcam1***	-0.779884	0.032783
NM_009910.2	***Cxcr3***	-3.309227	0.036463
NM_019732.2	***Runx3***	-3.035197	0.040985
NM_016960.1	***Ccl20***	-3.135620	0.041094
NM_011518.2	***Syk***	-0.654921	0.044742
NM_013532.2	***Lilrb4***	-0.822303	0.047020
NM_008355.2	***Il13***	-2.196345	0.049654
NM_008349.5	***Il10rb***	-0.493404	0.005406
NM_001037859.1	***Csf1r***	-0.542986	0.007283
NM_001025250.3	***Vegfa***	-0.577743	0.007533
NM_010798.2	***Mif***	-0.574122	0.008491
NM_010508.1	***Ifnar1***	-0.366953	0.009241
NM_009812.2	***Casp8***	-0.343917	0.009241
NM_007544.3	***Bid***	-0.583093	0.011645
NM_009982.2	***Ctsc***	-0.410117	0.011920
NM_009371.2	***Tgfbr2***	-0.395849	0.015089
NM_016923.1	***Ly96***	-0.477177	0.016295
NM_001113553.1	***Irak2***	-0.305006	0.018486
NM_008720.2	***Npc1***	-0.377297	0.018592
NM_008823.3	***Cfp***	-0.577043	0.020476
NM_013454.3	***Abca1***	-0.548182	0.020914
NM_011113.3	***Plaur***	-0.542416	0.022895
NM_011640.1	***Trp53***	-0.382943	0.028214
NM_009807.2	***Casp1***	-0.590112	0.029092
NM_009046.2	***Relb***	-0.365291	0.030723
NM_001163554.1	***Pou2f2***	-0.597801	0.035774
NM_001164735.1	***Crlf2***	-0.419599	0.036463
NM_009778.2	***C3***	-0.418029	0.036463
NM_010515.1	***Igf2r***	-0.284202	0.036463
NM_010549.3	***Il11ra1***	-0.512377	0.038363
NM_153098.3	***Cd109***	-0.402790	0.041094
NM_133990.4	***Il13ra1***	-0.419770	0.046369
NM_019777.3	***Ikbke***	-0.345539	0.047020
NM_001048177.1	***Jak2***	0.347616	0.032061
NM_011633.1	***Traf5***	0.360299	0.025435
NM_007987.2	***Fas***	0.341247	0.020486
NM_010578.1	***Itgb1***	0.385154	0.011645
NM_009848.3	***Entpd1***	0.388919	0.009241
XM_356827.6	***C7***	0.634353	0.009241
NM_008332.2	***Ifit2***	0.575927	0.007283
NM_011364.3	***Sh2d1a***	4.016440	0.006853
NM_008816.2	***Pecam1***	0.621827	0.003698
NM_015760.4	**Nox4**	0.682014	0.000903

Supplementary Table 3

Maximum values of carotid intima-media thickness (IMT) according to the *TREM1* SNP rs2234237 genotype in the 421 CoLaus substudy participants.

	AA	AT	TT	p-value ANOVA	p-value additive effect
N (% total)	340 (80.8)	75 (17.8)	6 (1.4)		
Left carotid					
Unadjusted	0.90±0.14	0.88±0.13	0.74±0.07	0.02	0.007
Adjusted [§]	0.90±0.01	0.89±0.02	0.75±0.05	0.02	0.006
Right carotid					
Unadjusted	0.85±0.15	0.82±0.12	0.77±0.05	0.14	0.14
Adjusted [§]	0.85±0.01	0.83±0.02	0.77±0.05	0.20	0.12

Results are expressed in mm and as mean ± standard deviation for unadjusted data or as adjusted mean ± standard error for multivariable-adjusted data. Statistical analysis by ANOVA and by linear regression supposing an additive genetic effect (qtlsnp command of Stata). [§] adjusted for gender, smoking status (never, former, current), age (continuous) and body mass index (continuous).

Supplementary Table 4

Clinical characteristics of the 421 CoLaus substudy participants according to their *TREM1* SNP rs2234237 genotype.

	**AA**	**AT**	**TT**	**P-value**
Sample size	340	75	6	
Women (%)	209 (61.5)	42 (56.0)	2 (33.3)	0.260
Age (years)	61.5 ± 5.3	60.6 ± 5.6	59.0 ± 5.2	0.253
BMI (kg/m^2)	26.2 ± 4.4	25.9 ± 4.0	27.2 ± 5.5	0.718
BMI categories (%)				0.776
Normal	145 (42.7)	34 (45.3)	3 (50.0)	
Overweight	132 (38.8)	28 (37.3)	1 (16.7)	
Obese	63 (18.5)	13 (17.3)	2 (33.3)	
Smoking (%)				0.602
Never	144 (42.4)	31 (41.3)	2 (33.3)	
Former	127 (37.4)	33 (44.0)	2 (33.3)	
Current	69 (20.3)	11 (14.7)	2 (33.3)	
Blood pressure (mm Hg)				
Systolic	142 ± 21	144 ± 19	148 ± 27	0.592
Diastolic	85 ± 12	87 ± 12	89 ± 13	0.260

BMI, body mass index. Results are expressed as number of participants (or percentage) or as average ± standard deviation. Between-group comparisons using Fisher's exact test for categorical variables or analysis of variance for continuous variables.

References

1. Choi, J.-H. *et al.* Flt3 signaling-dependent dendritic cells protect against atherosclerosis. *Immunity* **35,** 819–831 (2011).
2. Ensan, S. *et al.* Self-renewing resident arterial macrophages arise from embryonic CX3CR1+ precursors and circulating monocytes immediately after birth. *Nat Immunol* **17,** 159–168 (2015).
